# Transabdominal Preperitoneal (TAPP) versus Lichtenstein operation for primary inguinal hernia repair – A systematic review and meta-analysis of randomized controlled trials

**DOI:** 10.1186/s12893-017-0253-7

**Published:** 2017-05-10

**Authors:** Uwe Scheuermann, Stefan Niebisch, Orestis Lyros, Boris Jansen-Winkeln, Ines Gockel

**Affiliations:** Department of Visceral, Transplantation, Thoracic and Vascular Surgery, University Hospital of Leipzig, Liebigstrasse 20, 04103 Leipzig, Germany

**Keywords:** TAPP, Lichtenstein repair, Inguinal hernia, Outcome, Meta-analysis

## Abstract

**Background:**

Transabdominal Preperitoneal (TAPP) and Lichtenstein operation are established methods for inguinal hernia repair in clinical practice. Meta-analyses of randomized controlled studies, comparing those two methods for repair of primary inguinal hernia, are still missing. In this study, a systematic review and meta-analysis of published randomized controlled trials was performed to compare early and long term outcomes of the two methods.

**Methods:**

A literature search was carried out to identify randomized controlled trials, which compared TAPP and Lichtenstein repair for primary inguinal hernia. Outcome measures included duration of operation, length of hospital stay, acute postoperative and chronic pain, time to return to work, hematoma, wound infection, neuralgia, numbness, scrotal swelling, seroma and hernia recurrence. A quantitative meta-analysis was performed, using Odds Ratios (OR) or Standardized Mean Difference (SMD), and Confidence Interval (CI).

**Results:**

Eight controlled randomized studies were identified suitable for the analysis. The mean duration of the operation was shorter in Lichtenstein repair (SMD = 6.79 min, 95% CI, −0.68 – 14.25), without significant difference. Comparing both techniques, patients of the laparoscopic group showed postoperatively significantly less chronic inguinal pain (OR = 0.42; 95% CI, 0.23–0.78). Analyses of the remaining outcome measures did not show any significant differences between the two techniques.

**Conclusion:**

The results of this analysis indicate that complication rate and outcome of both procedures are comparable. TAPP operation demonstrated only one advantage over Lichtenstein operation with significantly less chronic inguinal pain postoperatively.

## Background

Inguinal hernia repairs are one of the most common operations in general surgery. Apart from the classical open repairs, minimally invasive approaches are increasingly preferred to manage groin hernia repair. However, the optimal surgical approach still remains controversial. The majority of the published studies, which aimed to compare the open with the minimal invasive operations for inguinal hernia repair, are non-randomized. Previous meta-analyses, which included the existed randomized controlled studies, provided insufficient differentiation between specific surgical techniques and patient characteristics [1–4]. Therefore, we aimed to provide a meta-analysis by including randomized controlled trials, which compared only one special laparoscopic repair (TAPP) with one open repair (Lichtenstein) technique in a predominantly homogenous subgroup of patients receiving primary hernia repair. We reviewed and compared systematically the outcomes after the two procedures with respect to operating time, acute postoperative and chronic inguinal pain, wound complications, intra- and postoperative complications, time to return to work, and hernia recurrence. To our knowledge, this meta-analysis is the first in which these approaches of hernia repair are compared.

## Methods

### Eligibility criteria and search

This meta-analysis follows the preferred reporting items for systematic reviews and meta-analyses (PRISMA) protocol [5]. In order to include all relevant studies comparing TAPP with the Lichtenstein technique for primary repair of inguinal hernia, research of the major data banks (PubMed, MEDLINE, Cochrane Library and ISRCTN (International Standard Randomized Controlled Trial Number)) was conducted. Randomized controlled trials, regardless of year of publication, number of cases, origin of hospital or country, have been included in this review. Registries have been searched for articles published up to July 2016 using the medical subject heading (MeSH) terms ‘inguinal hernia’, ‘groin hernia’, ‘TAPP’, ‘transabdominal’, ‘Lichtenstein’, ‘open hernia repair’ and ‘randomized’ in several combinations using the Boolean operators AND and OR.

### Inclusion and exclusion criteria

Studies with adult patients above 18 years of age of both genders who underwent inguinal hernia repair (direct and indirect) were included in the meta-analysis. Only published studies were used for the analysis. Studies, which included patients with recurrent inguinal hernias, irreducible scrotal hernia, femoral hernia or incarcerated hernia, requiring an emergency surgery were excluded. Non-randomized and non-controlled studies were also excluded. TAPP was performed with a three-port technique and the classical open Lichtenstein repair was performed as described before [6–8].

### Study selection

The identified studies were at first screened for duplicates. Titles and abstracts were then screened for trials that met the inclusion or exclusion criteria. After verifying the validity of the potential trial by reading the full-length article, data were extracted. Furthermore, the references from the included trials were searched to identify additional trials.

### Quality assessment

The included studies were evaluated for methodological quality using the guidelines of Jadad and colleagues [9].

### Data extraction

The following data were collected:

- Study characteristics: authors, year of publication, location of study, number of participating clinics, study period, other repair techniques included in the study, follow-up.
- Patient characteristics: number of patients, gender, age.
- Perioperative parameters: type of anesthesia, duration of operation, length of hospital stay.
- Outcome: acute postoperative pain, hematoma, seroma, wound infection, testicular atrophy, urinary retention, scrotal or genital neuralgia and numbness, scrotal or genital swelling, time to return to work, chronic inguinal pain, recurrence (whether it was reported early or late).

Only published data were used for the analysis. To investigate acute postoperative pain more exactly, Visual Analogue Scale (VAS), provided by the trials, were compared (0 indicates no pain and 10 or 100 severe pain). Chronic pain was defined as persistent inguinal pain three months after surgery. Hematoma, seroma and infection arising one month after the operation were considered to be wound complications. Postoperative complications included testicular atrophy, urinary retention, scrotal or genital neuralgia, numbness or swelling within one month after the operation.

### Statistical analysis

Statistical analysis was performed using the statistical software Review Manager Version 5.3 (Cochrane Collaboration, Oxford, UK). Forest plots displayed the relative strength of the treatment effects graphically. Studies that did not measure a particular parameter were excluded from the analysis. The Odds Ratio (OR) was calculated for binary data. Continuous variables were analyzed using the Standardized Mean Difference (SMD) to take into account the effect of the sample size. The 95% Confidence Interval (CI) was reported for each analyzed value. Heterogeneity was explored using the chi-square test, with the significance set at *P* < 0.05.

Similarly, I^2^ values were calculated to test for heterogeneity, with a value of 33% or less was considered to represent low heterogeneity. All outcomes were calculated with the random-effects model given the potential for heterogeneity in terms of the way and time point in which outcomes were assessed.

Where studies reported median and range instead of mean and variance, their mean and variance was calculated based on the methods described by Hozo and colleagues [10]. If the standard deviation was not available, it was calculated according to the guidelines of the Cochrane Collaboration [11].

## Results

### Eligible studies

**Fig. 1:**
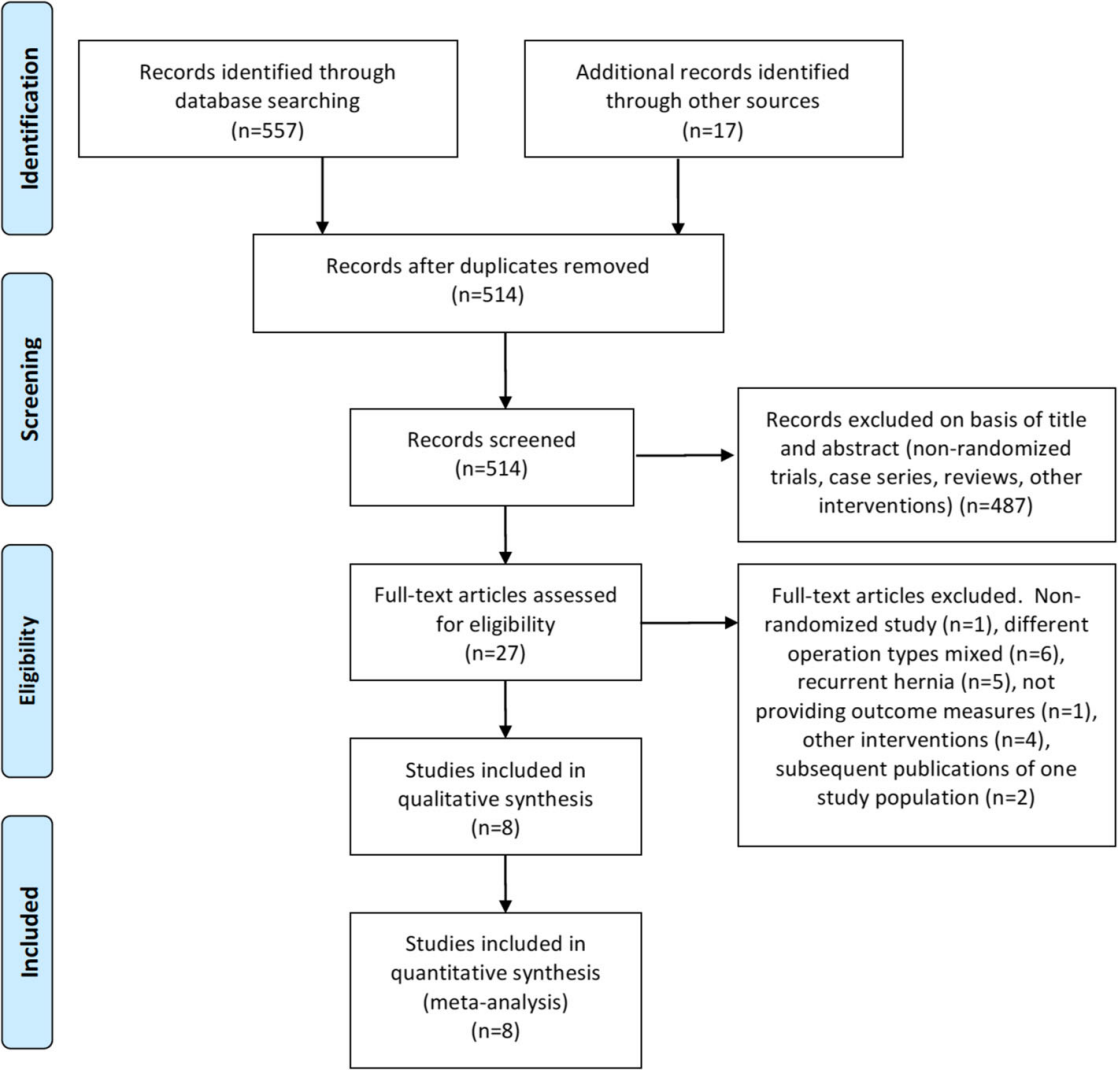
PRISMA flow chart for the selection of studies

Among 514 identified records, only eight were Randomized Controlled Trials (RCTs) directly comparing TAPP with Lichtenstein repair for primary inguinal hernia (Fig. 1) [12–21]. The three publications by Koeninger et al. [16, 17] and Butters et al. [18] described results of the same patient collective at different time points and were considered as one study. Of those, the study by Salma et al. [21] could not considered for the meta-analysis of postoperative complications and outcome due to its short mean follow-up, of only 36.9 h (Table 1). In total, 896 patients were included in the meta-analysis. Of those 425 received TAPP repair and 411 received a Lichtenstein repair for primary inguinal hernia. Polypropylene meshes were utilized either for TAPP or Lichtenstein tension-free hernia repair. For the TAPP group, in six trials, endoscopic staples were used for mesh fixation [12, 13, 15, 16, 19, 20], while in two trials the method of mesh fixation could not be determined [14, 21].

**Table 1:** Characteristics of included trials and patients

Author	Abbas et al. [12]	Anadol et al. [13]	Hamza et al. [14]	Heikkinen et al. [15]	Köninger et al. [16, 17]/ Butters et al. [18]	Picchio et al. [19]	Pokorny et al. [20]	Salma et al. [21]
Year	2012	2004	2010	1998	1998	1999	2008	2015
Country	Egypt	Turkey	Egypt	Finland	Germany	Latvia	Austria	Pakistan
Multicenter	no	no	no	no	no	no	yes	no
Period	May 2008-Sept 2011	N/A	N/A	Dec 1994-Jun 1995	Jul 1995-Jun 1996	Nov 1996-Dec 1997	1998–2002	Jan 2009 - Dec 2009
Other repair techniques	no	no	pre-peritoneal, TEP	no	Shouldice	no	Shouldice, Bassini, TEP	no
Number of patients								
Lichtenstein	97	25	25	20	93	52	69	30
TAPP	88	25	25	18	94	52	93	30
Age^a^, year, ±SD/ (range)								
Lichtenstein	34.6 ± 11.2	41.2 ± 10.9	35.12 ± 10.11	55.5^b^ (26–69)	53^b^ (26–74)	55.2 ± 12.4	52 (19–84)	N/A
TAPP	35.9 ± 12.104	41.8 ± 10.8	36.73 ± 12.06	51.0^b^ (34–68)	53^b^ (30–74)	57.7 ± 11.0	49 (21–78)	N/A
Gender (m/f)								
Lichtenstein	94/3	25/0	25/0	20/0	93/0	40/12	64/5	30/0
TAPP	86/2	25/0	25/0	17/1	94/0	37/15	86/7	30/0
Anesthesia	both: GA + LA	both: GA	N/A	open: LA, laparoscopic: GA	both: GA	open: LA, laparoscopic: GA	both: GA	N/A
F/U^a^, months (range)	17.9^b^ (8–30)	13.5 (8–28)	up to 24 weeks	17^b^	52^b^ (46–60) [16, 17]	1	up to 36	36.9 h
Jadad-Score	3	3	3	2	3	2	2	2

^a^All values are mean, except ^b^ median; [16–18] subsequent and supplementing publications; *N/A* not available, *TEP* Totally extraperitoneal endoscopic inguinal hernia repair, *SD* standard deviation, *m* male, *f* female, *LA* local anesthesia, *RA* regional anesthesia, *GA* general anesthesia, *F/U* Follow-up

The median Jadad-score for the included studies was two (range 2–3). The most common method of randomization was by computer generated random number allocation (three) [14, 19, 21], sealed envelopes (two) [15, 17], a central randomization service (one) [20] and random selection by balls (one) [13]. In one trial the method of randomization was not stated [12]. In six studies included in our meta-analysis, both operation methods were performed by a group of surgeons [12, 13, 16, 19–21]. In two studies both operation methods were performed by only one person [14, 15]. Experience of surgeons were described as skilled (six) [12–14, 16, 19, 20] or moderate (one) [15]. In one trial the experience could not be determined [21]. There were no significant differences in experience of surgeons performing the open and the laparoscopic interventions.

### Operation time

**Fig. 2:**
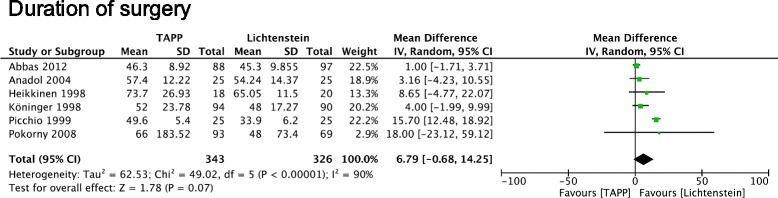
Forest plot of pooled mean difference with 95% CI for comparing TAPP with Lichtenstein hernioplasty, based on the assessment of operating time

Regarding the duration of the operation, all trials showed that the mean or medium time of operation in the TAPP group was longer than that in the Lichtenstein group. In the random-effects model (Fig. 2), the operation time was shorter in the Lichtenstein group with a mean difference of 6.8 min (95% CI, −0.68 – 14.25). Due to notable differences in operative times compared with the other trials the study by Hamza et al. was excluded from this meta-analysis. In this study, all operations were performed by one experienced surgeon [14]. Meta-analysis of subpopulations showed robust sensitivity and funnel plots revealed absence of publication bias (data not shown).

### Acute postoperative pain

Using Visual Analogue Scales (VAS), four trials reported quantitative measures of early and long-term postoperative pain [13, 14, 19, 21]. Pain within 12 h after surgery was investigated in three studies, in which the differences shown markedly favored the TAPP procedure (Table 2) [13, 14, 21].

**Table 2:** Early and long-term postoperative pain assessment of included trials using Visual Analogue Scale (VAS)

			VAS							
Author	Operation	VAS	0–12 h	p	12–24 h	p	24–48 h	p	48–72 h	p
Anadol et al. [13]										
	Lichtenstein	100-point	54.12 ± 13.06	<0.005	37.24 ± 11.38	<0.003	17.36 ± 4.52	NS	13.12 ± 5.95	NS
	TAPP		38.96 ± 8.21		20.92 ± 8.73		14.72 ± 7.03		9.44 ± 4.23	
Picchio et al. [19]										
	Lichtenstein	10-point	N/A	N/A	2.7 (range 1–5)	0.14	1.8 (range 1–4)	<0.03	N/A	N/A
	TAPP				3.1 (range 1–7)		2.3 (range 1–6)			
Salma et al. [21]										
	Lichtenstein	10-point	6.23 ± 1.87	0.005	N/A	N/A	N/A	N/A	N/A	N/A
	TAPP		4.43 ± 1.59							

All values are mean; *NS* not significant, *N/A* not available, *SD* standard deviation

### Postoperative complications

**Fig. 3:**
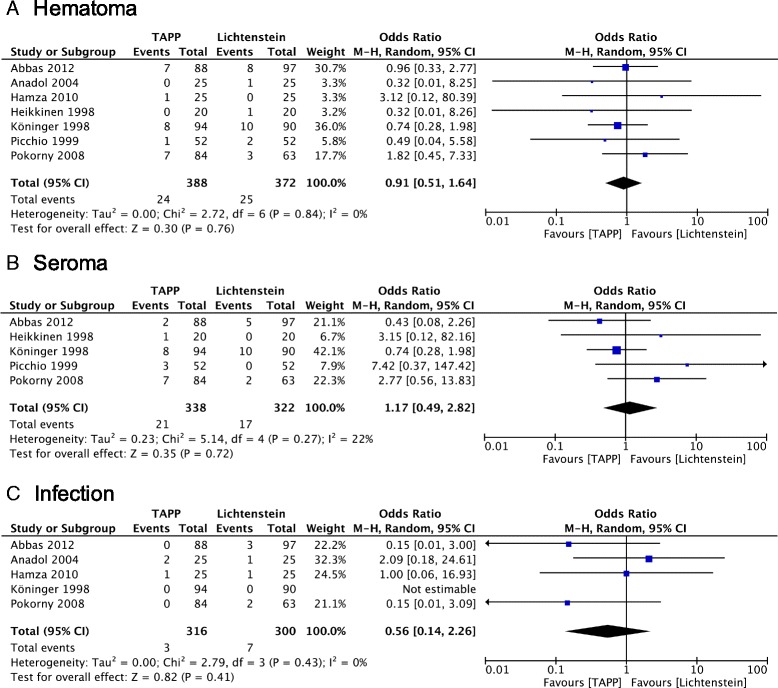
Forest plot of pooled odds ratio with 95% CI for comparing TAPP with Lichtenstein hernioplasty, based on the assessment of (**a)** hematoma, (**b)** seroma and (**c)** wound infection

**Fig. 4:**
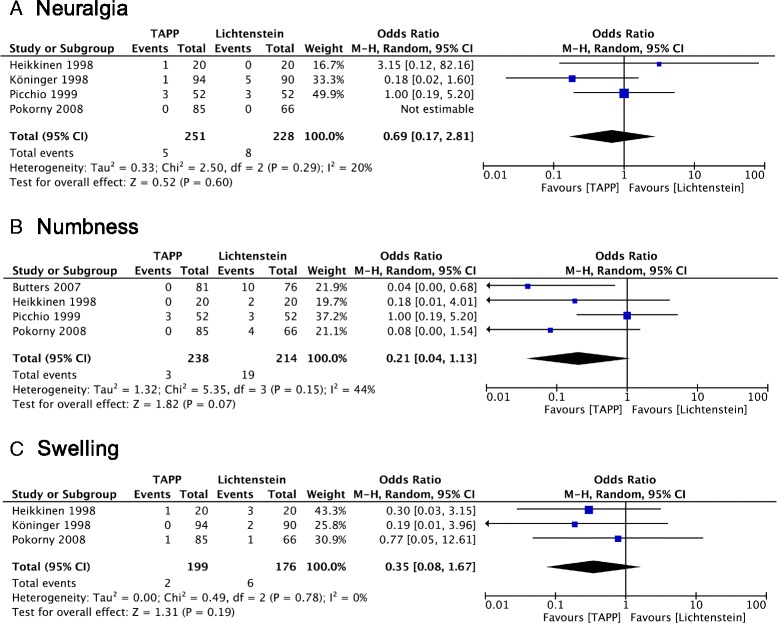
Forest plot of pooled odds ratio with 95% CI for comparing TAPP with Lichtenstein hernioplasty, based on the assessment of (**a)** neuralgia, (**b)** numbness and (**c)** swelling

The combined calculation showed no significant differences in terms of hematoma, seroma or infection after surgery between the two groups (*P* = 0.76, *P* = 0.72 or *P* = 0.41, respectively) (Fig. 3). Numbness was described in four trials and appear to be less common in patients receiving TAPP repair (*P* = 0.07). In the random-effects models, the risk of neuralgia and scrotal swelling were statistically similar between the two groups (*P* = 0.60; *P* = 0.19) (Fig. 4). Data of urinary retention and testicular atrophy were only available in one and two trials, respectively [16, 20] and no analysis was further performed.

Three out of seven trials reported serious intraoperative complications. An intraoperative hemorrhage occurred in eight patients out of 395 in the TAPP-group and in one patient out of 381 in the Lichtenstein repair group [19, 20]. Following TAPP repair, injury of the viscus and intestinal obstruction arose in one patient each [12]. Due to intraoperative complication, four patients in the TAPP group the minimal invasive approach had to be converted to an open procedure [12, 14, 19]. No study reported deep wound infection or infection of the mesh. No correlation was found between the experience of the surgeon and rate of serious complications. All interventions with serious complications were performed by groups of well-trained surgeons.

### The length of hospital stay and return to work

In the included trials, the mean duration of hospital stay ranged from 3.5 h to 5 days (data not shown). The length of hospital stay was excluded from our meta-analysis due to different hospital policies, health care systems and the unknown status of employment of the included patients.

**Fig. 5:**
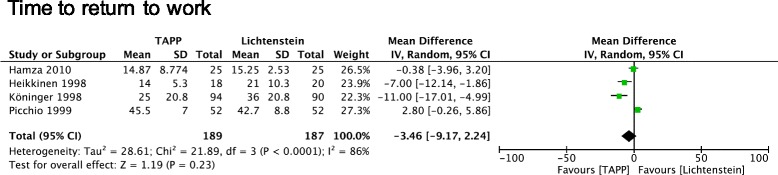
Forest plot of pooled odds ratio with 95% CI for comparing TAPP with Lichtenstein hernioplasty, based on the assessment of time before return to work

Three out of four trials reported details for “time to return to work” and supported that the median time to return to work was longer in the Lichtenstein group. However, in the random-effects model no statistical difference was found between both groups (Fig. 5) (SMD = −3.46 days, 95% CI, −9.17 – 2.24).

### Chronic Pain

**Fig. 6:**
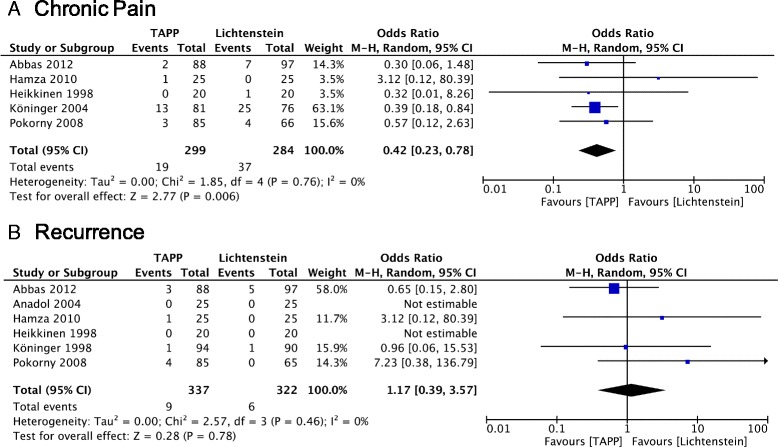
Forest plot of pooled odds ratio with 95% CI for comparing TAPP with Lichtenstein hernioplasty, based on the assessment of **a** chronic pain and **b** recurrence

Five studies provided information on chronic pain postoperatively (Fig. 6a). There was no heterogeneity (*P* = 0.76, I^2^ = 0%) among trials. In the random-effects model (OR = 0.42; 95% CI, 0.23–0.78), there was a significant difference in terms of chronic pain after surgery between the two groups.

### Hernia recurrence

Six trials reported details of hernia recurrence. In the random-effects analysis of 337 patients who underwent TAPP and 322 patients who underwent Lichtenstein repair (Fig. 6b), there was no significant difference (OR = 1.17; 95% CI, 0.39–3.57).

## Discussion

Randomized controlled trials (RCT) comparing TAPP with Lichtenstein repair for primary inguinal hernia are rare and mostly represent limited patient numbers. Thus, we performed a meta-analysis by identifying suitable studies for a better assessment of the relative merits of each surgical technique.

### Operation time

Longer operation duration is translated in protracted anesthesia and higher procedure costs. Our analysis confirmed previous studies [1–3], which supported that operation time is shorter in Lichtenstein group compared with that in the TAPP group, however without showing significant difference. The study of Hamza et al. showed significant differences in operation time compared with the other studies. This individual peculiarity may be a result of the low number of study participants. Furthermore, in most studies included in our meta-analysis, both operation methods were performed by a group of surgeons making results more homogeneous. Laparoscopic approach is technically more difficult and intraperitoneal conditions such as adhesions, could explain delays seen in TAPP procedures. However, in case of bilateral hernias, endoscopic approach seems to be of advantage with respect to the length of operation [22].

### Acute postoperative pain

Next to age and hernia recurrence [23], preoperative and early postoperative pain seems to be risk factors for chronic inguinal pain syndrome following hernia repair [24–26]. Notably, most studies lack assessment of inguinal pain either in the preoperative or the postoperative phase. Furthermore, the severity of pain along with the request on pain medication in the postoperative course are poorly documented. Based on the reported data, by comparing data of VAS (Visual Analogue Scales) we revealed differences in pain scores within 12 h postoperatively [13, 14, 21], in favor of TAPP procedure. Of note, the different time points of the VAS-documentation and the various types of anesthesia make conclusions in favor of one technique over the other very difficult. Although, all patients in the TAPP group received general anesthesia, in two trials of this meta-analysis patients undergoing open repair received local anesthesia [15, 19]. Remarkably, in the study by Salma et al. [21], all patients either in TAPP group or Lichtenstein group received additionally to the general anesthesia also local anesthesia.

### Postoperative complications

Complications were infrequent in both groups. Although, previous studies comparing endoscopic techniques (TAPP and TEP) with open, tension-free operations, revealed significantly lower incidence of wound infection and hematoma together with higher incidence of seromas after endoscopic repair [1, 3], our meta-analysis shows no similar differences. Different follow-up time points and prevalence in describing postoperative complications reduces methodological quality of the study. As noted by Schmedt et al. [3], hematomas after endoscopic repair might stay clinically unnoticed. In the trials included in our meta-analysis, no case of mesh infection or deep wound infection was reported. In line with other studies, genital or scrotal numbness was less common after TAPP procedures [1, 22, 24]. An explanation for this may be an intraoperative ilioinguinal or genitofemoral nerve injury in the course of the open approach.

Even if the complication rates are low and significant differences between the two groups could not be revealed [1–3], serious intraoperative complications following TAPP, which may include serious visceral or vascular injuries should be mentioned [12, 19, 20].

### Return to work

In most studies, patients returned earlier to work after TAPP repair than after Lichtenstein operation, without, however the differences to reach significance (*P* = 0.23) [14–18]. Previous sub-population meta-analyses showed a significant shorter convalescence period after endoscopic repair [2, 3]. However, these meta-analyses did not distinguish between different endoscopic (TAPP and TEP) or open hernia repair (Lichtenstein and non-Lichtenstein) techniques.

### Chronic Pain

The laparoscopic approach reduces the risk of chronic pain. In a large prospective study comparing 244 patients after Lichtenstein with 198 patients after laparoscopic TAPP repair, Aasvang et al. [24] showed that the incidence of persistent post-hernioplasty pain was significantly lower in TAPP (8.1%) versus with Lichtenstein repair (16%). In line with this, our meta-analysis also shows a significant difference in terms of chronic pain after surgery between the groups (*P* = 0.006). One explanation is the increased tissue damage of open surgery. While in Lichtenstein repair the spermatic cord and the cremaster muscle have to be dissected. During TAPP procedure, the pain most likely is caused by the dissection of the parietal peritoneum. In support of the latter, Bansal et al. [25] associated TAPP repair with a significantly higher incidence of early postoperative pain compared to Totally Extraperitoneal (TEP) hernia repair, due to the incision of peritoneum.

### Hernia recurrence

Comparing the TAPP and Lichtenstein operations, our meta-analysis showed no significant difference in terms of hernia recurrence (*P* = 0.46). Previous sub-population meta-analyses comparing endoscopic approaches (TAPP and TEP), with open tension-free techniques revealed a higher recurrence rate following endoscopic repair [2, 3]. Schmedt et al. attributes this to a higher number of TEP repairs included in their meta-analysis [3, 27]. The recurrence rate--especially in endoscopic repairs--depends on the experience of the surgeon [1, 7, 28, 29]. Most TAPP surgeons were described as skilled [12–14, 16, 19, 20], explaining the low recurrence rates in our study among other parameters.

## Conclusion

Our meta-analysis showed that TAPP is especially associated with significantly less chronic inguinal pain in comparison with Lichtenstein repair. No further significant differences were found between the two methods, but moderate methodological quality and low number of patients of the included studies making further multicenter trials necessary.

## Abbreviations

CI: Confidence Interval
ISRCTN: International Standard Randomized Controlled Trial Number
MeSH: Medical subject heading
OR: Odds Ratios
PRISMA: Preferred Reporting Items for Systematic Reviews and Meta-Analyses
RCT: Randomized Controlled Trial
SMD: Standardized Mean Difference
TAPP: Transabdominal Preperitoneal
TEP: Totally Extraperitoneal
VAS: Visual Analogue Scale
